# Acute Exacerbation of Interstitial Lung Disease: A Case Series and a Narrative Literature Review

**DOI:** 10.3390/arm94030042

**Published:** 2026-06-22

**Authors:** Bartłomiej Czyżak, Adam Lasota, Sebastian Majewski

**Affiliations:** Department of Pneumology, Medical University of Lodz, 90-153 Lodz, Poland

**Keywords:** acute exacerbation, interstitial lung disease, AE-ILD, idiopathic pulmonary fibrosis, antifibrotics

## Abstract

**Highlights:**

**What are the main findings?**

- Acute exacerbation (AE) of interstitial lung disease (ILD) is a life-threatening condition with no confirmed effective treatment.
- Most data on AE-ILD are of low quality and are derived from small observational studies.

**What are the implications of the main findings?**

- High-quality prospective studies with large sample sizes are needed to determine effective treatment strategies for AE-ILD.
- Antifibrotics play a major role in AE-ILD prevention.

**Abstract:**

Acute exacerbation of interstitial lung disease (AE-ILD) represents sudden, severe deterioration in patients with pre-existing ILD and is associated with high morbidity and mortality. Our work presents a case series of AE-ILD in patients with idiopathic pulmonary fibrosis (IPF), idiopathic non-specific interstitial pneumonia (iNSIP), and connective tissue disease-associated ILD (CTD-ILD) managed at our institution and provides a narrative review of AE-ILD. Across cases, AE-ILD manifested as rapid progression of dyspnea and extensive ground-glass opacities (GGOs) on imaging, often triggered by infections or immune-mediated processes. Despite treatment, all cases were fatal, confirming that mortality remains high in AE-ILD. In our literature review, we focus on dysregulated innate immunity, an altered microbiome, potential microaspiration, surgical procedures, and autoantibody-mediated inflammation as triggers, as well as the risk factors for and prevalence of AE-ILD. We also examine pharmacological and non-pharmacological interventions, with particular emphasis on the role of antifibrotic agents as a key protective factor. Evidence for and against corticosteroid use in AE-IPF and non-IPF AE-ILD is discussed, highlighting the radically different treatment approach for AE in melanoma differentiation-associated gene 5 (MDA5)-positive dermatomyositis (DM)-associated ILD compared to AE-IPF. Our findings underscore the heterogeneous presentation and poor prognosis of AE-ILD, emphasizing the urgent need for standardized diagnostic criteria, risk stratification, and prospective studies with larger cohorts to establish evidence-based therapeutic strategies.

## 1. Introduction

Interstitial lung disease (ILD) is an umbrella term for heterogeneous conditions grouped due to similarities in clinical presentation, pulmonary function impairment, and diffuse parenchymal lung involvement on imaging studies [1]. Prolonged inflammation of the lung parenchyma, which often occurs in ILD, may lead to fibrosis and destruction of lung architecture. The most common ILDs include idiopathic pulmonary fibrosis (IPF), connective tissue disease-associated ILDs (CTD-ILDs), chronic hypersensitivity pneumonitis (HP), and sarcoidosis [1]. The natural history of ILDs may be punctuated by sudden episodes of disease deterioration called acute exacerbations (AEs). AE is defined as the rapid worsening of respiratory symptoms of <1 month duration with new ground-glass opacities (GGOs) and/or consolidations visualized on computed tomography (CT), in the absence of heart failure and fluid overload [1,2]. Due to a scarcity of clinical data, the exact incidence of AE-ILD is unknown; however, it is estimated that the annual incidence of AE ranges from 4 to 14% in IPF patients [3]. Furthermore, many studies have indicated that AE-IPF occurs more frequently than AE in non-IPF ILDs [1]. The pathogenesis of AE-ILD is still uncertain, but it is suspected that factors such as infection, aspiration, pulmonary emboli, and mechanical stretch of the lung parenchyma may influence its development [1,4]. AE-ILD is a life-threatening event, with AE-IPF associated with the worst prognosis; in-hospital mortality reaches approximately 50%, and the median survival is 3 to 4 months [2]. Unfortunately, there is no evidence supporting the effectiveness of any therapy for this life-threatening condition, which places patients and physicians in a challenging situation [1]. In this article, we present a case series of AEs in different types of ILD encountered at our institution and discuss the available literature on AE-ILD.

## 2. Case Reports

### 2.1. Patient 1

**Figure 1:**
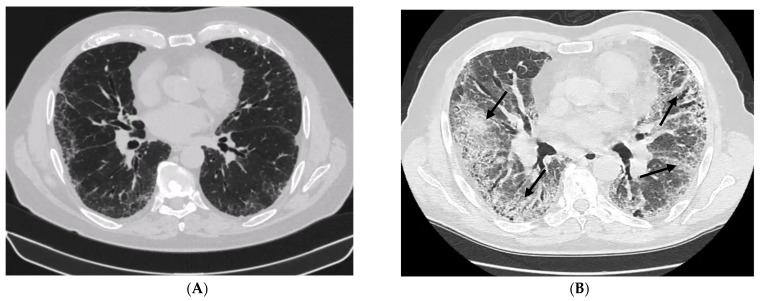
(**A**) HRCT of an IPF patient at baseline, showing a UIP pattern. (**B**) HRCT of an IPF patient with bilateral GGO and consolidations superimposed on the UIP pattern corresponding to the clinical diagnosis of AE-IPF. Arrows indicate newly developed consolidations and GGOs superimposed on a UIP pattern.

A 72-year-old male was admitted for evaluation of a chronic cough that had lasted 18 months. The patient denied exertional dyspnea, chest pain, weight loss, and reduced exercise capacity. The patient’s medical history included Parkinsonism, cholelithiasis, dyslipidemia, benign prostatic hyperplasia, an incisional hernia at the appendectomy site, a history of smoking (15 pack-years), and occupational exposure in the mining and steel industries. On physical examination, bilateral crackles were auscultated over the lower lung fields. Cardiac examination revealed a regular sinus rhythm at a rate of 82 beats per minute (bpm), and blood pressure (BP) was 156/82 mmHg. Pulmonary function tests (PFTs) were normal: forced vital capacity (FVC), 4.0 L (111% predicted); forced expiratory volume in 1 s (FEV1), 3.57 L (130% predicted); diffusing capacity of the lung for carbon monoxide (DLCO), 5.91 millimoles per minute per kilopascal (mmol/min/kPa), 91% of the predicted value; and peripheral oxygen saturation (SpO_2_), 95% without oxygen supplementation. High-resolution computed tomography (HRCT) revealed a usual interstitial pneumonia (UIP) radiologic pattern (Figure 1A). After excluding known causes of fibrosis, IPF was diagnosed during a multidisciplinary team (MDT) discussion involving a pulmonologist, radiologist, and rheumatologist. The patient was enrolled in the antifibrotic treatment program and started on nintedanib 150 mg twice daily. At the first follow-up visit, one month after the initial admission, laboratory tests revealed elevated liver enzyme levels, with alanine aminotransferase (ALT) at 145 U/L and aspartate aminotransferase (AST) at 126 U/L (normal value < 38 U/L), which necessitated temporary discontinuation of the medication. At the subsequent visit two weeks later, hepatic enzyme levels had returned to normal values, and nintedanib was reintroduced at a reduced dose of 100 mg twice daily. After 3 months of antifibrotic treatment, the patient was admitted to the Department of Pneumology in moderately severe general condition, with fever, exacerbation of cough, dyspnea, and lower-limb edema. Auscultation revealed bilateral crackles, predominantly in the lower lung fields, with additional involvement of the mid-lung zones. The patient was tachycardic, with a heart rate (HR) of 106 bpm. Symmetrical edema of the lower extremities, extending to the mid-calves, was noted. Laboratory tests revealed white blood cell (WBC) count: 14 × 10^3^ cells/µL; C-reactive protein (CRP): 54.5 mg/L; and respiratory failure based on arterial blood gas (ABG) analysis—pH: 7.4; pressure of carbon dioxide (pCO_2_): 45.8 mmHg; pressure of oxygen (pO_2_): 50.2 mmHg; concentration of bicarbonate (HCO_3_): 27.5 millimole/L: and SpO_2_: 86%. HRCT revealed reticular changes with thickening of the interlobular and intralobular septa, traction bronchiectasis, and bilateral peripheral GGO, mostly located in the lower lobes, with no fluid in the pleural cavity (Figure 1B). Based on the clinical presentation, a diagnosis of AE-IPF, likely triggered by infection, was established. The patient received a single intravenous (IV) dose of hydrocortisone 100 mg, followed by oral methylprednisolone therapy with gradual tapering. Additional broad-spectrum antibiotic therapy—amoxicillin with clavulanic acid and clarithromycin—intensification of diuretic treatment, and oxygen therapy using a non-rebreather mask (NRM) at a flow rate of 10–12 L/min were initiated. After one week, clinical improvement allowed discharge from the hospital on home oxygen therapy (HOT) via nasal cannula at 3 L/min, with an SpO_2_ of 91% at rest. Three weeks later, following pulmonary rehabilitation, the patient was readmitted due to worsening dyspnea. The patient’s general condition was severe, with bilateral crackles over the lung fields below the inferior angle of the scapulae, an HR of 120 bpm, and an SpO_2_ of 70% without oxygen supplementation. The laboratory tests showed a WBC count of 14 × 10^3^/cells/µL, a CRP level of 13.9 mg/L, and ABG findings indicating type I respiratory failure: pH, 7.44; pCO_2,_ 39.7 mmHg; and pO_2,_ 41.5 mmHg. HRCT findings were similar but more pronounced. The patient received three IV pulses of methylprednisolone, each at a dose of 1000 mg, followed by oral prednisone, ceftriaxone, and morphine. Oxygen therapy at a flow rate of 8–10 L/min using an NRM was initiated, which maintained SpO_2_ at 93%. After two weeks and clinical stabilization, the patient was discharged with HOT at up to 10 L/min. Ten days later, he was readmitted via emergency medical services due to worsening dyspnea. During transport, the patient experienced respiratory arrest; however, spontaneous breathing resumed after approximately one minute of resuscitation. In the department, desaturation to an SpO_2_ of 60% prompted initiation of continuous positive airway pressure (CPAP), with distending pressure to the lungs set at 14 cm of water (cmH_2_O) and a fraction of inspired oxygen (FiO_2_) of 100%. Over the following hours, respiratory exhaustion progressed, leading to sudden cardiac arrest (SCA). Considering the poor prognosis, resuscitation was withheld.

### 2.2. Patient 2

**Figure 2:**
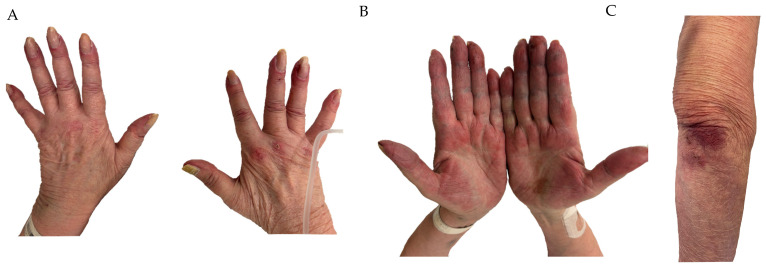
(**A**) Gottron’s sign. (**B**) Inverse Gottron’s sign. (**C**) Inflammatory lesions over the left elbow.

A 72-year-old woman was admitted to the Internal Medicine Department due to a non-productive cough, dyspnea on exertion, and general malaise. She did not report fever, night sweats, chest pain, muscle weakness, or unintentional weight loss. Her medical history included cholecystectomy, hypercholesterolemia, and a 20-pack-year smoking history. Moreover, her mother died due to an undefined lung disease. On admission, crackles were audible at the bases of both lungs, and SpO_2_ without oxygen was 87%. The initial chest X-ray revealed bilateral filling of the alveoli with a tendency to consolidation. However, after initial clinical diagnosis of community-acquired pneumonia (CAP), treatment with broad-spectrum antibiotics and oxygen therapy with a nasal cannula, the patient showed no improvement. In the next step, CT angiography of the chest excluded pulmonary embolism and showed bilateral GGOs and thickening of interlobular septa. Despite low levels of inflammatory parameters (WBC: 4.8 × 10^3^/µL; CRP: 9.85 mg/L; procalcitonin (PCT): 0.12 ng/mL), the patient’s condition deteriorated. Therefore, she was transferred to the Department of Pneumology for further diagnosis and treatment. On admission, the patient presented with dyspnea at rest, increased respiratory effort, hypoxemia (SpO_2,_ 74%, despite NRM with 12 L/min oxygen therapy), and partly compensated respiratory alkalosis based on ABG analysis: pH, 7.5; pCO_2,_ 23.7 mmHg; HCO_3,_ 8.3 mmol/L; pO_2,_ 47.1 mmHg. The patient had cyanosis of the palms and yellow spots on the fingernails, accompanied by characteristic cutaneous manifestations of dermatomyositis, including Gottron’s papules and inverse Gottron’s sign (Figure 2A,B). In addition, inflammatory skin lesions were also observed over the elbows (Figure 2C). Detailed medical history revealed the presence of ulcers and wounds of the metacarpophalangeal joints for about six months. Therefore, underlying, previously undiagnosed and untreated CTD was suspected. A panel of serum autoantibodies was performed, revealing high levels of melanoma differentiation-associated gene 5 (MDA5) and Ro-52 antibodies. Consequently, a diagnosis of clinically amyopathic dermatomyositis (CADM) was established with AE-ILD presentation. After excluding infection, treatment with pulses of methylprednisolone IV and noninvasive high-flow nasal oxygen therapy (HFNOT), with 30 L of O_2_ per minute and systematic increase to 60 L/min. Over the next two days, a slow decline in levels of inflammatory parameters was observed; however, respiratory failure progressed, which led to sudden cardiac arrest and death of the patient.

### 2.3. Patient 3

**Figure 3:**
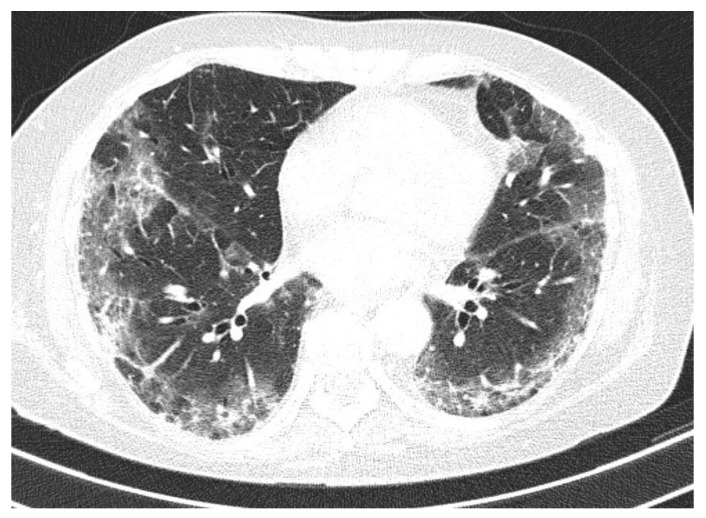
HRCT of a patient with AE-ILD in the course of RA-ILD.

A 66-year-old woman was admitted to the hospital due to worsening dyspnea, cough, low-grade fever, and general weakness. The patient’s medical history included rheumatoid arthritis (RA), hyperthyroidism, diabetes mellitus type 2 (DM2), and occasional cardiac supraventricular beats. The patient was diagnosed with RA four years before admission due to pain and swelling of the talocrural and metacarpophalangeal joints, and RA-ILD was identified one year later, based on an HRCT scan that revealed mild honeycombing and small adjacent GGO areas. RA was treated with methotrexate and prednisolone. The patient complained of progressive exertional dyspnea and cough for about a year, with a weekly onset of worsening symptoms. She denied chest pain, weight loss, or hemoptysis. On auscultation, she had bilateral crackles in the lower lung fields. Moreover, laboratory tests did not indicate a suspected infection: WBC, 6 *×* 10^3^/cells/µL; CRP, 1 mg/L. ABG analysis initially showed no abnormalities: pH, 7.42; pCO_2_, 37.2 mmHg; pO_2_, 86.7 mmHg; HCO_3_: 23.5 millimole/L, SpO_2_: 96%. HRCT scan performed on admission demonstrated persistent mild honeycombing in both lung bases, consistent with irreversible fibrotic changes, which were also present on a CT scan taken approximately one year earlier. However, significant progression of interstitial abnormalities in both lungs was evidenced by the presence of extensive GGOs affecting all lobes bilaterally (Figure 3). Due to the worsening symptoms, a bronchoscopy was performed, revealing mucopurulent bronchitis. Bronchoaspirate showed positive polymerase chain reaction (PCR) results for *Pneumocystis jiroveci* and Cytomegalovirus (CMV). Although there were no typical signs of infection, it was suspected that an opportunistic infection might have triggered the AE-ILD. Antimicrobial therapy with trimethoprim-sulfamethoxazole and antiviral therapy with ganciclovir were initiated. Gradual and rapid worsening of respiratory failure was observed despite escalating oxygen therapy. Within a week of admission, the patient was transferred to the Intensive Care Unit (ICU), where she was sedated and intubated, and mechanical ventilation was initiated in Volume Control–Assist Control (VC-AC) mode with 70% FiO_2_ and a positive end-expiratory pressure (PEEP) of 9 cmH_2_O. Another CT scan was performed, which revealed significant progression of interstitial changes in both lungs. Immunoglobulins were added to the therapy; however, the patient presented no signs of recovery. Finally, cyclophosphamide was added to the therapy. Despite broad-spectrum antimicrobial and immunosuppressive therapy, the patient presented with disseminated intravascular coagulation (DIC) and multi-organ failure (MOF) that led to sudden cardiac arrest and death, 15 days after admission to the hospital.

### 2.4. Patient 4

A 64-year-old male was admitted to the Department of Pneumology for evaluation of ILD. The patient reported periodic worsening of cough and an itchy rash, which had first appeared five years earlier. HRCT of the chest revealed honeycombing, thickening of the interlobular and intralobular septa, areas of GGO, and mildly increased traction bronchiectasis in the basal segments of both lower lobes. Past medical history included hypertension, degenerative joint disease, glaucoma, and a history of smoking (10 pack-years).

Physical examination revealed slightly audible crackles at the bases of both lungs, more pronounced on the right side, and small crusted skin lesions on the right leg, which had been present for approximately five years. SpO_2_ was 97% without oxygen therapy. PFT and 6-minute walking test (6MWT) were performed; the patient walked 400 m without desaturation. FVC was 73% of the predicted value with no signs of obstruction, and DLCO was 91% of the predicted value. During the next hospitalization, four months later, for further investigation, bronchoscopy with transbronchial lung cryobiopsy (TBLC) and bronchoalveolar lavage (BAL) were performed. BAL cytology revealed 5.3% neutrophils, 84.7% macrophages, 8% lymphocytes, and 2% eosinophils. PFT values showed slight progression: FVC, 75% of predicted; DLCO, 71% of predicted. During the 6-minute walking test (6MWT), the patient walked 440 m, with oxygen desaturation ranging from 98% to 91%. Serologic tests were performed. Anti-nuclear antibodies (ANAs), anti-neutrophil cytoplasmic antibodies (ANCAs), and the scleroderma panel were negative. The myositis panel was positive for OJ, and ubiquitin-like modifier activating enzyme (SAE) antibodies and anti-cyclic citrullinated peptide (anti-CCP) antibodies were elevated at 154 U/mL. After the MDT discussion, a working diagnosis of fibrosing non-specific interstitial pneumonia (fNSIP) with a probable RA-related cause was established. However, the patient did not fully meet the American College of Rheumatology/European Alliance of Associations for Rheumatology (ACR/EULAR) criteria (elevated anti-CCP, no joint involvement; 4 points). MDT recommended establishing the final diagnosis after histopathological evaluation of TBLC samples and postponing antifibrotic treatment until completion of the diagnostic process.

**Figure 4:**
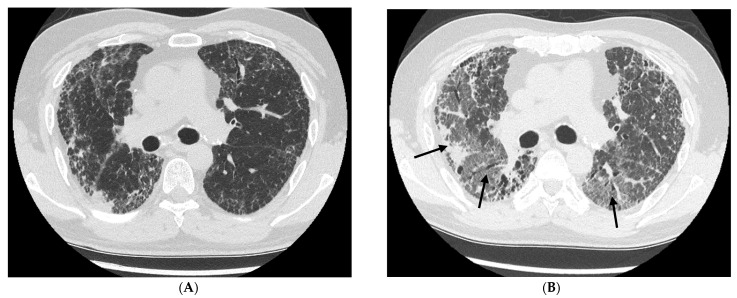
(**A**) HRCT of patient 4 with fNSIP pattern before AE-ILD. (**B**) HRCT of patient 4 with fNSIP pattern during AE-ILD. Arrows indicate newly developed consolidations and GGOs superimposed on a fNSIP pattern.

One year after the first admission, the patient was readmitted to the Department of Pneumology for further evaluation. PFTs indicated slight progression—FVC, 67% of predicted; DLCO, 55% of predicted—and desaturation during 6MWT worsened from 97% to 85% after 450 m. HRCT demonstrated significant progression of pulmonary fibrosis with the appearance of consolidations in the right upper lobe (Figure 4A). Histopathological examination revealed dilatation of the bronchial lumen with fibrotic thickening of the wall, hypertrophy of smooth muscle, and moderate lymphocytic infiltration with occasional developing lymphoid follicles; however, it did not indicate a specific diagnosis. Due to deterioration in FVC, radiological progression, and clinical worsening, a diagnosis of progressive fibrosing interstitial lung disease (PF-ILD) was established, and the patient was approved for antifibrotic treatment with nintedanib. MDT considered the introduction of anti-inflammatory treatment after positron emission tomography-computed tomography (PET-CT) in order to exclude neoplastic disease. Following PET-CT exclusion of a neoplastic process, prednisone and mycophenolate mofetil were prescribed.

Two years after the first admission and seven months after initiation of nintedanib therapy, the patient was admitted to our center due to rapidly progressive dyspnea. On physical examination, the patient was conscious and in logical contact. SpO_2_ was 88% on 10 L/min passive oxygen therapy. Oxygen therapy was escalated to HFNOT at 60 L/min, with a SpO_2_ of 96%. Auscultation revealed crackles over all lung fields, most pronounced at both lung bases, with slightly diminished vesicular breath sounds over the right lung base and tachycardia of 117/min. Peripheral edema was absent. Laboratory tests showed elevated D-dimer (1450 ng/mL), lymphopenia (300 cells/µL), elevated CRP (115 mg/L) with negative procalcitonin, a normal WBC count, mild hyponatremia, and elevated B-type natriuretic peptide (BNP, 3376 pg/mL), with no evidence of acute myocardial injury (AMI). CT of the chest revealed diffuse GGOs and consolidations superimposed on fibrotic changes. Empirical treatment with amoxicillin-clavulanic acid and clarithromycin was initiated. Additionally, sulfamethoxazole-trimethoprim and pulse therapy with 500 mg methylprednisolone were administered, and mycophenolate mofetil was discontinued. Bronchoscopy was performed with bronchoaspirate sampling for PCR testing, which revealed Influenza A virus and presence ofspecific genes for Methicillin-resistant *Staphylococcus aureus* (MRSA). Consequently, oseltamivir was initiated, and antibiotic therapy was modified to vancomycin and clarithromycin. Despite therapy, the patient required progressive intensification of oxygen therapy; however, he refused non-invasive ventilation (NIV) due to poor tolerance. Because of desaturation to 70% and extreme respiratory effort, the patient was transferred to the ICU. During ICU treatment, he required endotracheal intubation, mechanical ventilation, and deep sedation. Despite mechanical ventilation, severe hypercapnia up to pCO_2_ 120 mmHg persisted, together with signs of extreme respiratory failure, and the circulatory system required support with norepinephrine infusion. After consultation with a transplantology center, the patient was disqualified from lung transplantation due to his critical condition. In the absence of causal treatment options, a do-not-resuscitate (DNR) order was implemented, and palliative care was initiated. The patient died after seven days of hospitalization, including two days in the ICU.

## 3. Discussion

### 3.1. Definition and Classification of AE-ILD

AE-ILD refers to a rapid, clinically significant respiratory deterioration occurring in patients with pre-existing ILD or with new-onset ILD complicated by AE. However, there is no universally accepted definition of AE-ILD; the diagnostic criteria established for AE-IPF are commonly applied in clinical practice. The definition of AE-IPF has evolved over time. The most recent international working group report from 2016 defines AE as rapid clinically significant respiratory deterioration characterized by new or worsened dyspnea and evidence of new, widespread alveolar abnormalities that developed over less than one month. A previous or concurrent diagnosis of IPF must be established. The widespread alveolar abnormalities must be confirmed by a CT scan, which reveals new bilateral GGOs and/or consolidations superimposed on a background consistent with the UIP pattern. The clinical presentation should not be fully explained by cardiac failure or fluid overload. AE can be classified as triggered (e.g., infection, aspiration, post-procedural, or postoperative) or idiopathic. Clinical events that are considered AE-IPF, but do not completely fulfill diagnostic criteria due to a missing CT scan, should be termed “suspected AE-IPF” [2]. The new classification of interstitial pneumonias (IPs) is based on the pathobiology of ILD, placing AE-ILD in a new group of entities—diffuse alveolar damage (DAD)—characterized by rapid progression and its corresponding histological and radiological morphology. This group includes idiopathic DAD (iDAD), former acute interstitial pneumonia (AIP), and secondary causes (secondary DAD), both of which clinically manifest as acute respiratory distress syndrome (ARDS). Histologically, DAD is characterized by three phases: an exudative phase, defined by thickening of alveolar septa due to interstitial edema and pneumocyte hyperplasia, with alveoli covered by hyaline membranes; an organizing phase, marked by proliferation of loose connective tissue and atypical pneumocytes; and a fibrotic phase, which may result from progression of the organizing phase and corresponds to residual fibrosis and potential ongoing progression. Radiologically, DAD manifests as patchy or diffuse GGOs that may progress to fibrosis over time, with significant traction bronchiectasis, architectural distortion, and honeycombing. DAD is the predominant histopathological pattern in AE-ILD, but other manifestations, including alveolar hemorrhage, organizing pneumonia (OP), or nonspecific inflammatory changes, can also be observed. Given this inconsistency, Ryerson et al. suggest that AE-IPF should have a revised definition based on pathobiology that includes specific HRCT and clinical criteria to identify DAD and distinguish AE from other conditions [1,4,5].

### 3.2. RP-ILD Controversy

AE-ILD should not be confused with rapid progressive ILD (RP-ILD), which is a recognized entity in the literature; however, it has not yet been formally included in the American Thoracic Society (ATS)/European Respiratory Society (ERS) classification of ILD. In most cases, RP-ILD is associated with CTD-ILD, particularly IIM with MDA5 antibodies. MDA5 is associated with CADM, characterized by the presence of anti-MDA5 antibodies and cutaneous inflammatory manifestations, including heliotrope rash, Gottron’s papules, Gottron’s sign, inverse Gottron’s sign, and rashes at the inner canthus and auricle. A hallmark feature of MDA5-positive CADM is the high frequency of RP-ILD, occurring in 50–90% of patients [6]. Importantly, an official consensus definition of RP-ILD has not been established. In most studies, it is defined by either worsening dyspnea or progression on HRCT within three months [7]. In MDA5-positive patients, RP-ILD can manifest with a variety of patterns on HRCT, including OP, OP with NSIP, or nonspecific parenchymal changes, including GGO, reticular opacities, or consolidations [6]. The definition of RP-ILD overlaps with that of AE-ILD; therefore, in some cases, this entity may also be classified as AE-ILD. Therefore, a precise definition of RP-ILD is essential for clinical and research purposes.

### 3.3. The Pathobiology of AE-ILD

Patients with IPF are particularly susceptible to respiratory infections. Macrophages derived from the lungs of patients with IPF exhibit impaired antigen recognition, phagocytosis, and antigen presentation capacities [8]. Another feature observed in IPF patients is the reduced expression of co-stimulatory molecules, which further impairs the adaptive immune response [8,9]. Nevertheless, existing data do not support the role of infection as a key trigger in the development of AE-IPF. In a study by Oda et al., the autopsies of 52 AE-IPF patients showed that only 28.8% of patients had bronchopneumonia [10]. Furthermore, a study by Wootton et al. found that only 3 out of 43 patients with AE-IPF were infected with common respiratory viruses, such as parainfluenza viruses and coronaviruses, as indicated by the presence of viral ribonucleic acid (RNA) in BAL fluid. However, 15 patients were infected with non-respiratory viruses, including Herpes simplex virus (HSV), Epstein–Barr virus (EBV), and Torque Teno virus (TTV) [11]. In AE-IPF, an increased bacterial burden has been observed, along with alterations in the respiratory microbiome, including an increase in *Campylobacter* species and *Stenotrophomonas* species, and a decrease in *Veillonella* compared to stable IPF, suggesting that even when a specific microbial pathogen cannot be identified, the infection may contribute to AE development [12,13]. Moreover, in non-IPF ILDs, infection is more often considered a trigger for AE. In a study by Zhang et al., approximately half of AEs in 69 patients with idiopathic inflammatory myopathy (IIM) associated with ILD were triggered by infection [14]. Loss of lung parenchyma and immune function in fibrotic areas may also lead to alterations in the microbiome and dysfunctional host–microbiome interactions, which, over time, may act as a sustained source of injurious stimuli and promote AE development [8,13]. Another potential cause of pulmonary epithelial injury includes microaspirations resulting from gastroesophageal reflux (GER), which may trigger the development of AE-IPF. The study by Lee et al. detected pepsin in BAL fluid from patients with AE-IPF, which supports a role for gastric microaspirations in these events [15]. The limited available data on the effectiveness of anti-acid therapy in preventing AE-ILD is inconsistent. A pooled analysis of the INPULSIS trials showed that patients receiving anti-acid therapy had a higher incidence of AE-IPF in the placebo group, while the incidence was comparable among those treated with nintedanib [16]. However, to clearly define the role of anti-acid therapy in preventing AE, large randomized controlled trials (RCTs) specifically addressing this intervention are required.

In AE, injury from various causes to the alveolar epithelium and vascular endothelium leads to the release of cytokines and chemokines, which recruit neutrophils and monocyte-derived macrophages to the damaged lung tissue. These recruited cells secrete proinflammatory cytokines, such as interleukin-6 (IL-6), interleukin-23 (IL-23), and tumor necrosis factor-alpha (TNF-α), which promote Th17 cell differentiation. The resulting cytokine milieu, characterized by elevated levels of IL-17 and IL-23, further stimulates epithelial cells and fibroblasts to secrete additional cytokines, thereby amplifying the inflammatory response. The infiltration and activation of neutrophils within the pulmonary parenchyma contribute directly to acute lung injury. During AE-IPF, lymphocyte activity is markedly suppressed, while the innate immune response predominates. Consequently, corticosteroid therapy primarily targets lymphocyte proliferation and activity, with limited efficacy on innate immune mechanisms, which may explain the limited effectiveness of these drugs in the treatment of AE [8]. Furthermore, the chronically fibrotic lung environment sensitizes immune cells to hyperinflammatory responses. Transforming growth factor-beta (TGF-β), a key profibrotic cytokine, has been shown to synergize with pathogen recognition receptor activation, thereby amplifying inflammatory signaling [8,17]. This immunopathogenic interplay may explain the observed positive association between antifibrotic therapy and improved clinical outcomes in patients experiencing AE-IPF. One hypothesis involves B lymphocytes and autoantibodies. Compared to healthy individuals, patients with IPF show increased levels of B lymphocytes in both the peripheral blood and lung compartments. This may be due to exposure to autoantigens released during lung injury, such as heat shock protein (HSP) and annexin-1. Immunization with these autoantigens triggers the production of autoantibodies, which contribute to enhanced inflammation. It has been found that these autoantibodies have higher concentrations during AE-IPF compared to stable disease [8,18,19,20].

### 3.4. Incidence and Risk Factors of AE-ILD

The lack of a universally accepted definition, together with the varied course and severity of ILDs, complicates case reporting and precludes precise determination of AE-ILD incidence. Studies indicate that AE in IPF patients is significantly more frequent than in non-IPF patients [21]. Several studies have assessed the incidence of AE in IPF patients, most of which were conducted retrospectively. The meta-analysis by Wang et al., which included 11,855 IPF patients, reported an annual AE incidence ranging from 4% to 14%, with an overall incidence of 9%. The incidence of AE increased over time, reaching 13% within 2 years and 19% within 3 years of follow-up [3]. Prospective studies are even more limited. The study by Tsubouchi et al. prospectively evaluated 528 idiopathic interstitial pneumonia (IIP) patients, including 306 with IPF, during a 5-year follow-up. AE-IPF occurred in 21.6% of cases, and 33.3% of AE-IPF patients experienced multiple AE episodes (range, 2–5 episodes) [22]. There are limited data on the exact incidence of AE in other forms of ILD. The annual incidence of AE in CTD-ILD patients ranges from 1.25% to 3.3%, and in NSIP, it is approximately 4.2% [23,24]. In RA-ILD, the annual incidence varies from 5.6% in the overall population to 11.1% in RA-ILD patients with a UIP pattern [23]. The higher number of AEs observed in RA-ILD patients compared to the general CTD-ILD population may be explained by the fact that the UIP pattern is associated with an increased risk of AE-ILD and is the most common pattern seen in RA [24]. The risk factors for AE-ILD include poor lung function, recent FVC decline, previous AE, pulmonary hypertension, coronary artery disease, infections, aspirations, or invasive procedures such as lung resection, surgical lung biopsy (SLB), cryobiopsy, and even BAL. Furthermore, the pneumotoxicity from various medications or radiation therapy may induce AE development, as observed in patients treated with chemotherapy and radiotherapy [2,4,25].

### 3.5. Mortality of AE-ILD

AE-ILD is associated with major implications for the patient’s prognosis. The absence of effective treatment strategies for patients with AE-ILD is closely associated with poor survival outcomes. Retrospective studies indicate that AE-IPF is associated with the worst prognosis. Up to 46% of deaths of IPF patients are preceded by AE; the in-hospital mortality of AE-IPF patients is around 50%, and the median life expectancy of survivors ranges between 3 and 4 months [2]. In the previously mentioned prospective study by Tsubouchi et al., the 3-month mortality after an AE episode was 39.4%; AE was the second most common cause of death (23.9%), following chronic respiratory failure (34.9%) [22]. Although AE-ILD in non-IPF patients has better outcomes, AE remains life-threatening [1]. There is a gap in knowledge regarding the exact mortality, particularly in non-IPF ILDs. In a retrospective study from Japan, 90-day mortality after AE was significantly higher in IPF patients (57%) compared to non-IPF IIPs (29%) and CTD-ILD patients (33%) [26]. In a more recent study that retrospectively assessed 133 patients with progressive pulmonary fibrosis (PPF), the 1-, 3-, and 5-year mortality rates in the AE group were 21.4%, 65.6%, and 86.2%, respectively [27]. In a systematic review and meta-analysis of 35 studies, Pitre et al. identified several prognostic factors associated with mortality in AE-ILD. Poor outcomes were associated with IPF compared with non-IPF ILD, long-term supplemental oxygen therapy at baseline, higher CT scores, a diffuse pattern on HRCT, invasive mechanical ventilation (IMV), corticosteroid use before hospital admission, a longer interval from admission to treatment initiation, and an increased percentage of neutrophils in BAL. In contrast, FVC, DLCO, and serum lactate dehydrogenase (LDH) levels were not significantly associated with an increased risk of death [28]. In a cohort study by Ba et al., higher serum neutrophil percentage was associated with increased in-hospital mortality of AE-ILD patients, while pulmonary hypertension (PH) was identified as a negative prognostic factor only in the AE-IPF subgroup [29]. Infection as a trigger of AE is also considered an unfavorable prognostic factor. In a retrospective cohort study by Zhang et al. on AE-ILD in an IIM cohort of patients (*n* = 69), 30-day, 90-day, and 1-year mortality rates were significantly elevated in the infection-related AE group compared with the idiopathic AE group—28.6%, 34.3%, and 54.3% vs. 5.9%, 14.9%, and 17.6%, respectively (*p* = 0.002) [14]. However, an IIM represents only a subset of CTDs; therefore, larger studies are needed to determine whether these findings are generalizable to other CTD-ILD populations.

### 3.6. Pharmacological Treatment of AE-ILD

#### 3.6.1. Corticosteroid Therapy

Currently, there is no evidence that any available therapy significantly improves prognosis in AE-IPF patients. The 2016 working group report suggests that corticosteroid use in AE-IPF patients may be beneficial; however, this recommendation is considered weak due to the low-quality, anecdotal evidence [2]. The 2011 ATS/ERS/Japanese Respiratory Society (JRS)/Latin American Thoracic Association (ALAT) Clinical Practice Guidelines recommend that the majority of AE-IPF patients be treated with corticosteroids; however, this recommendation is also considered weak, as it is based on very low-quality evidence and has not been revised in the subsequent updates from 2015 and 2022 [30,31,32]. The most recent meta-analysis including 19 studies with 3277 AE-IPF patients showed that corticosteroid treatment increases mortality (relative risk (RR) of 1.78; confidence interval (CI) 1.29–2.76, *p* = 0.00001). The greatest limitation of this systematic review and meta-analysis is that many of the included studies did not control for disease severity when comparing steroid-treated patients to controls. This raises the possibility that more severely ill patients were more likely to receive steroids, which could have contributed to the observed higher mortality in the steroid-treated group [33]. Another important issue is the relationship between the dose of corticosteroids and patient outcomes, which was not analyzed in this work. In a study that addressed this issue, an initial low dose of corticosteroids (0.5 mg/kg/day), maintained after corticosteroid pulses, was associated with better 3-month survival compared to a higher initial dose (1 mg/kg/day). However, this study has similar limitations to those discussed above. Patients treated with the higher corticosteroid dose (1 mg/kg/day) were likely in worse condition [34]. There is consideration that high doses of corticosteroids might improve survival in non-IPF patients after an AE episode. A retrospective study by Jang et al. evaluated 117 patients with AE-IPF and 65 patients with non-IPF AE-ILD, comparing outcomes between high (>1 mg/kg) and low corticosteroid doses (0 to 1 mg/kg). While no survival benefit was observed in AE-IPF, the administration of high-dose corticosteroids in AE patients with non-IPF ILDs was associated with improved 90-day survival [35]. Future RCTs with improved matching methods are needed to clarify the impact of steroids on outcomes in AE-IPF and AE in non-IPF patients. Currently, one clinical trial assessing high-dose corticosteroid pulse therapy in AE-IPF is ongoing (NCT05674994), whereas another has been completed without published results (NCT04996303).

#### 3.6.2. Combination of Corticosteroids and Immunosuppressants

Faced with a lack of effective treatment, an alternative approach to treating AE has been to add another immunosuppressant to corticosteroid therapy. Some small-sample observational studies suggest that adding tacrolimus or cyclosporin may improve outcomes in patients with AE-IPF [36,37]. Furthermore, studies investigating autoantibody-targeted therapies are currently underway. An ongoing phase III trial is evaluating the efficacy of intravenous immunoglobulin (IVIG) in patients with AE-IPF (NCT07299695). In addition, a phase I/II trial enrolled 11 patients with AE-IPF who were treated with plasma exchange, rituximab, and IVIG, demonstrating significantly improved 1-year survival compared with historical controls [38]. Building on these findings, two RCTs (NCT03584802 and NCT03286556) are evaluating the efficacy of plasma exchange combined with rituximab and IVIG in patients with AE-IPF. Adjunctive use of immunosuppressive agents alongside high-dose corticosteroids can also result in unfavorable outcomes. A phase III RCT included 119 patients with AE-IPF, who were randomized into an intervention group receiving corticosteroids combined with cyclophosphamide and a control group receiving corticosteroids with a placebo. The addition of cyclophosphamide to corticosteroid therapy in AE-IPF patients was associated with increased 3-month mortality compared to corticosteroid monotherapy, 45% (27/60) vs. 31% (18/59), respectively—a difference of 14.5%; however, this difference was not statistically significant (95% CI −3.1 to 31.6, *p* = 0.10) [39]. Moreover, previous immunosuppression may be a risk factor for mortality in AE. In a retrospective study by Papiris et al., patients with AE-IPF who had never received immunosuppressive therapy had significantly better outcomes. One-year survival was 65% in the non-immunosuppressed group versus 17% in those with a history of immunosuppression, despite similar supportive treatment [40].

#### 3.6.3. Human Recombinant Thrombomodulin

It is speculated that AE-IPF is associated with pulmonary vascular endothelial cell injury caused by inflammation and impaired pulmonary microcirculation attributed to microthrombi induced by coagulopathy. These data provided the basis for another potential therapy: recombinant human soluble thrombomodulin (thrombomodulin alfa), an approved treatment for DIC known for its anti-inflammatory effects [41]. The phase 3 RCT published in 2020 involved 80 AE-IPF patients, who were randomized into two groups to receive high-dose corticosteroid therapy with placebo or with thrombomodulin alfa (380 U/kg/d for 14 days by intravenous infusion). The addition of thrombomodulin alfa did not improve the 90-day survival compared to the placebo group and was associated with a higher risk of bleeding. The authors concluded that the use of thrombomodulin alfa should not be recommended for AE-IPF treatment [42].

#### 3.6.4. Antimicrobial Treatment

In addition to high-dose corticosteroids, broad-spectrum antibiotics are commonly used in the management of AE-IPF. However, in the working group report on AE-IPF, there are no recommendations on the appropriate use or management of antimicrobial therapy in these patients [2]. There is evidence suggesting that azithromycin may be beneficial in the treatment of AE-ILD. Kawamura et al., in a cohort of 85 AE-IPF patients, retrospectively compared outcomes related to azithromycin and fluoroquinolone-based regimens. The results showed that treatment with azithromycin was associated with better prognosis compared with fluoroquinolone therapy (60-day mortality 26% vs. 70%, respectively) [43,44]. Studies investigating the immunomodulatory effects of azithromycin in AE-IPF are currently underway. An ongoing RCT (NCT05842681) is evaluating the efficacy of azithromycin in combination with pirfenidone compared with placebo plus pirfenidone in patients with AE-IPF. Dysregulated immunity in IPF is associated with alterations in the respiratory microbiome. IPF patients exhibit an imbalance of airway microbiota, characterized by increased bacterial and viral load [8]. Although chronic antibiotic therapy with co-trimoxazole was hypothesized to modify disease outcomes in an RCT by Wilson et al., the study did not demonstrate any improvement in prognosis or reduction in hospitalization rates, which indirectly reflects AE incidence [45]. Despite the unfavorable results of co-trimoxazole, some evidence suggests that the use of macrolides may have a protective role against AE-IPF. In a small observational retrospective study with a 36-month follow-up, only 4 of 29 patients (13.8%) treated with macrolides experienced AE, compared to 8 of 23 patients (34.8%) in the non-macrolide group (*p* = 0.047). Furthermore, macrolide administration was significantly associated with better survival [46]. Although these results are encouraging, they need to be confirmed in larger prospective studies.

#### 3.6.5. Antifibrotic Therapy

##### The Role of Antifibrotics in AE-ILD Prevention

Antifibrotic therapy is approved for slowing the disease progression, but it also seems to hold promise in reducing AE frequency. The post hoc analysis of the phase III RCTs ASCEND and CAPACITY showed that pirfenidone treatment was associated with a reduction in respiratory-related hospitalizations during the study year (7% vs. 12%; hazard ratio (HR), 0.52; 95% CI, 0.36–0.77; *p* = 0.001). Unfortunately, AE-IPF was not a predefined endpoint in these studies [47]. In a meta-analysis by Wu et al., nine RCTs with 1011 IPF patients receiving pirfenidone and 912 controls receiving placebo were summarized; the analysis showed that treatment with pirfenidone was associated with a lower risk of AE, with an RR (risk ratio) of 0.64 (95% CI 0.49–0.84, I^2^ = 7%) [48].

A pooled analysis of TOMORROW and INPULSIS studies demonstrated that treatment with nintedanib, in addition to slowing disease progression, was associated with a prolonged time to first AE-IPF (HR: 0.53, 95% CI: 0.34–0.83). Furthermore, patients treated with nintedanib experienced fewer episodes of multiple AEs than those in the placebo group (4.6% vs. 8.7%, respectively) [49]. Moreover, in a pooled analysis of INPULIS trials, the frequency of AE was significantly lower in the nintedanib group compared to placebo. The incidence rate of AE was 2.2 per 100 patient-years in the nintedanib group and 5.8 per 100 patient-years in the placebo group (RR, 0.37; 95% CI 0.19, 0.72; *p* = 0.003) [50]. In the INBUILD trial (Efficacy and Safety of Nintedanib in Patients with Progressive Fibrosing Interstitial Lung Disease), a reduced risk of AE was observed in the subgroup with a UIP-like fibrotic pattern, in contrast to the findings in the overall population [51]. Recently, nerandomilast, a phosphodiesterase-4 (PDE4B) inhibitor with both antifibrotic and immunomodulatory properties, has been introduced for the treatment of IPF and PF-ILD. The pivotal registration trials, FIBRONEER-IPF and FIBRONEER-ILD, evaluated two groups of patients with IPF and PF-ILD. Secondary endpoints in these trials included time to first AE of IPF or non-IPF ILD, hospitalization for a respiratory cause, or death, as well as the composite endpoint of AE or death. Significant benefits were observed only in the PF-ILD population (FIBRONEER-ILD study). Treatment exclusively with the higher dose of nerandomilast (18 mg) was associated with a significantly lower risk of AE-ILD or death (HR, 0.59; 95% CI, 0.41–0.84). In addition, a trend toward a reduced risk for first AE, respiratory hospitalization, or death was observed (HR, 0.77; 95% CI, 0.59–1.01; *p* = 0.06), although this result did not reach statistical significance [52,53].

##### Role of Antifibrotics in Post-Operative AE Prevention

Invasive procedures are a strong risk factor for AE-ILD development. However, perioperative use of antifibrotics might help prevent postoperative AE-IPF. In a retrospective study by Bongiolatti et al., 55 patients with IPF and lung cancer (LC) who underwent LC resection surgery were analyzed and divided into two groups: those treated with antifibrotic therapy (either chronic or prophylactic use; n = 29), the majority (83%) of whom received pirfenidone, and those without such treatment (n = 26). The incidence of postoperative AE was significantly lower in the antifibrotic group compared with the non-treated group (3.4% vs. 23.1%, *p* = 0.044). Moreover, antifibrotic therapy was identified as a strong protective factor against postoperative AE in multivariable analysis. There were no significant differences in 30-day or 90-day mortality between the groups [54]. In another study by Iwata et al. (phase II trial), 36 IPF patients received pirfenidone and subsequently underwent LC resection surgery. Only one patient (2.8%) experienced an AE. However, the study had a small sample size and lacked a non-pirfenidone control group [55]. Consequently, a large-scale phase III trial (UMIN000029411) is currently underway to further evaluate these findings. Moreover, the efficacy and safety of perioperative nintedanib administration remain unclear. The drug’s label does not recommend its use in the perioperative period due to potential interference with the wound-healing process.

##### Role of Antifibrotics After AE Development

Another important question is whether antifibrotic treatment should be continued or initiated during an AE event. There is some evidence that an antifibrotic agent might improve the survival of patients who have already developed AE. A retrospective study from South Korea, which included 1213 IPF patients, found that treatment with an antifibrotic agent was associated with a lower incidence of AE (12.9% vs. 21.9%) and reduced mortality following an AE event (HR, 0.60; 95% CI, 0.42–0.85; *p* = 0.004). The median survival duration after AE was 4 months in the antifibrotic group and 1 month in the no-antifibrotic group (*p* < 0.001). Furthermore, the administration of antifibrotics was also associated with lower all-cause mortality, all-cause hospitalizations, and respiratory-related hospitalizations. In this study, the date of initiation of antifibrotic therapy was not assessed [56]. A different approach was applied in a national database cohort study from Japan by Urushiyama et al., who investigated the use of nintedanib with an unspecified dosage to AE-ILD patients initiated within 14 days post-admission (n = 353), compared to patients without nintedanib (n = 5882) treatment. The authors found significantly lower in-hospital mortality rates in the nintedanib group, with rates of 6% for nintedanib versus 13.7% for the control group (odds ratio (OR), 0.43; 95% CI, 0.27–0.70; *p* < 0.001). Moreover, nintedanib administration was associated with shorter duration of hospitalization, 30.7 ± 13.7 days for nintedanib versus 37.5 ± 19.0 days for the control group (*p* < 0.001) [57]. The data from these studies are promising; however, large randomized prospective studies are necessary to confirm the aforementioned findings.

#### 3.6.6. Pulmonary Hypertension Treatment as an AE-ILD Prevention

Pulmonary hypertension is a risk factor for AE-ILD development and a poor prognostic factor in AE-ILD episodes [4,29]. The prevalence of PH in ILD increases with disease severity, ranging from 3.5 to 15% in early disease to 60–90% in patients listed for lung transplantation. PH-ILD is usually mild to moderate; therefore, severe PH should prompt evaluation for alternative causes of PH [58]. The classification of PH in CTD-ILD is particularly challenging. Although group 1 pulmonary arterial hypertension (PAH) and group 3 PH-ILD are the predominant phenotypes, they may occur separately or coexist in the same patient. Importantly, no clear threshold of ILD extent has been established to reliably distinguish group 1 PAH from group 3 PH-ILD. This distinction is clinically relevant because, until recently, PAH-targeted therapies were primarily reserved for patients with group 1 PAH [59]. Data on the distribution of PH groups in CTD-ILD remain limited. In one cohort of patients with systemic sclerosis, 55% had group 3 PH, 24% had group 1 PH, and 21% had group 2 PH [60]. The use of pulmonary vasodilators in patients with parenchymal lung disease has long been controversial because of concerns regarding worsening gas exchange and potential harm [59]. However, the INCREASE trial demonstrated the efficacy of inhaled treprostinil in PH-ILD, leading to its approval for PH treatment in group 3 [61]. Accordingly, the 2022 European Society of Cardiology (ESC)/ERS guidelines recommend inhaled treprostinil for patients with PH-ILD and suggest phosphodiesterase-5 (PDE-5) inhibitors in selected patients with severe PH [62]. It is important to note that, in the INCREASE trial, fewer patients in the inhaled treprostinil group experienced exacerbations of their underlying ILD compared with the placebo group (26.4% vs. 38.7%, respectively) [61]. In the open-label extension study of the INCREASE trial, Waxman et al. assessed outcomes up to 108 weeks after the completion of the 16-week trial. The time to AE of underlying lung disease was significantly prolonged in patients who had previously received inhaled treprostinil compared with those who had previously received a placebo (HR, 0.69; 95% CI, 0.49–0.97; *p* = 0.0321), corresponding to a 31% reduction in the risk of AE. Similarly, inhaled treprostinil was associated with a significantly lower risk of AE of underlying lung disease (HR, 0.70, 95% CI, 0.49–0.99; *p* = 0.0437) [63]. However, in the most recent RCTs, TETON-1 and TETON-2, which demonstrated the antifibrotic potential of inhaled treprostinil, no significant difference was observed between the treprostinil and placebo groups in the time to first AE-IPF (HR, 0.50; 95% CI, 0.23–1.08) [64]. Therefore, treprostinil may have a potential role in AE-ILD prevention. Nevertheless, current evidence remains insufficient to draw definitive conclusions, and further studies are needed to determine which ILD subgroups are most likely to benefit from this effect and the duration of treatment required for such benefits to become clinically apparent.

#### 3.6.7. MDA5 Treatment

RP-ILD, similarly to AE-ILD, is associated with poor prognosis in patients with MDA5-positive DM. A meta-analysis by Yang et al. assessed 15 studies comprising a total of 1153 MDA5-positive DM-ILD patients. Among these, three studies included RP-ILD in the analysis. RP-ILD was identified as a significant risk factor for mortality in MDA5-positive DM-ILD patients (HR = 4.02, 95% CI: 1.89–8.55, *p* < 0.001) [65].

However, the clinical course of AE-ILD differs from that of RP-ILD in MDA5-positive patients. In this setting, RP-ILD is associated with a high mortality risk during the first six months; however, when treatment is initiated early and proves effective, long-term outcomes can be favorable, in contrast to the generally poor prognosis of AE-ILD [6]. Risk factors for poor outcomes include older age, presence of skin ulcers, hypoxemia, advanced lung disease, and elevated levels of CRP, creatine kinase (CK), LDH, ferritin, and anti-MDA5 titers, as well as the coexistence of anti-Ro52 antibodies [6].

In MDA5-positive DM, the therapeutic strategies based on corticosteroids, either as monotherapy or as part of step-up regimens with subsequent immunosuppressive agents, have demonstrated only limited efficacy; however, triple immunosuppressive therapy (high doses of glucocorticoids, tacrolimus, and cyclophosphamide) administered concurrently with corticosteroids appears to be more effective [6]. In a single-arm prospective study from Japan, 29 patients with new-onset MDA5-positive DM with ILD were enrolled. They were treated with a regimen of high-dose glucocorticoids, tacrolimus, and intravenous cyclophosphamide. Plasmapheresis was used if a patient’s condition worsened after initiation of the regimen. The data were compared with a historical control group consisting of 15 anti-MDA5-positive DM patients with ILD who received step-up treatment (high-dose glucocorticoids and stepwise addition of immunosuppressants). The group treated with a combined immunosuppressive regimen showed significantly higher 6-month survival rates than the step-up treatment group (89% versus 33%; *p* < 0.0001) [66]. Moreover, others have reported that triple immunosuppressive therapy as induction treatment may prevent relapses and facilitate drug-free remission [6]. In a retrospective study from Japan, 68 MDA5-positive DM-ILD patients were followed after the initial admission for RP-ILD. After 10 years, the recurrence-free survival in the group receiving triple therapy was 90%, compared with 56% in the conventional treatment group receiving glucocorticoid monotherapy or a single immunosuppressant (*p* = 0.02). Furthermore, the subgroup in which triple therapy was initiated earlier also showed better recurrence-free survival compared with the group in which triple therapy was administered later (92% vs. 83%, respectively); however, the difference was not statistically significant (*p* = 0.06). Consequently, treatment with triple therapy was associated with a higher rate of drug withdrawal and drug-free remission compared with the conventional treatment group [67]. It is also worth noting that the available data suggests that plasmapheresis, IVIG, Janus kinase (JAK) inhibitors, and rituximab may improve outcomes in cases of non-responders to triple immunosuppressive therapy [18]. For example, in the previously mentioned study, 6-month survival rates were even higher in patients receiving additional plasmapheresis compared with triple immunosuppressive therapy alone (89% vs. 71%, respectively) [66]. However, available data are derived from small sample retrospective studies; therefore, there is an urgent need to confirm these observations in well-designed and properly conducted prospective studies. According to the most recent ERS and EULAR guidelines for the treatment of IIM-ILDs, corticosteroids, calcineurin inhibitors (CNIs), and rituximab are strongly recommended regardless of disease severity, based on the available evidence. In cases of RP-ILD, the guidelines provide a conditional recommendation for a combination of high-dose corticosteroids, cyclophosphamide, and rituximab or CNI. Additionally, IVIG, JAK inhibitors, and plasmapheresis may be considered [68]. This represents a radically different recommendation compared to AE-IPF treatment, where accumulating evidence challenges the effectiveness of steroid use [1].

### 3.7. Non-Pharmacological Strategies of AE Treatment

#### 3.7.1. Hemoperfusion with Polymyxin B-Immobilized Fibers

Direct hemoperfusion with polymyxin B (PMX-DHP) has been shown to improve oxygenation in patients with acute lung injury (ALI) or ARDS and may also benefit outcomes in septic shock. By extracorporeally adsorbing and removing inflammatory cytokines, PMX-DHP has prompted investigations into its potential role in AE-ILD [69,70]. Small, retrospective studies have suggested that PMX-DHP might be beneficial in patients with AE-IPF. One such study was conducted by Onishi et al., who assessed 50 patients with AE-IPF, all of whom received corticosteroid pulse therapy. Among them, 27 patients underwent PMX-DHP, which was identified as a significant predictor of survival (HR = 0.442, 95% CI 0.223–0.873; *p* = 0.019). Furthermore, the 12-month survival rate was significantly higher in the PMX-DHP group than in the non-PMX group (41.7% vs. 9.8%; *p* = 0.04) [70]. Recently, a multicenter, single-arm prospective study from Japan enrolled 20 patients with AE-IPF who received PMX-DHP therapy. Treatment with PMX-DHP was associated with improvements in the arterial oxygen partial pressure to fraction of inspired oxygen (PaO_2_/FiO_2_) ratio and alveolar–arterial oxygen partial pressure difference, as well as a decrease in CRP levels compared to baseline. The 1-month survival rate was approximately 65%, and the 12-week survival rate was 50% [71]. However, larger prospective studies are necessary to validate the therapeutic effectiveness of PMX-DHP in AE-IPF.

#### 3.7.2. Non-Invasive Support of Ventilation

AE manifests as rapid respiratory deterioration; therefore, conventional oxygen therapy (COT) is often required for these patients. However, in more severe cases, COT may be insufficient, and ventilatory support becomes necessary. In extreme cases, IMV may be required. Although IMV is associated with 90% in-hospital mortality, the recommendation for its use is weak [2]. To avoid IMV, there is a need for strategies that can improve ventilation without increasing risk, such as non-invasive ventilation (NIV), which can be divided into two modalities: NIPPV and HFNOT. In a meta-analysis by Sanguanwong et al., the use of NIV procedures (NIPPV and HFNOT) was associated with a significant improvement in the PaO_2_/FiO_2_ ratio compared to COT. There were no differences in mortality or intubation rates between HFNOT and NIPPV [72]. Due to its better tolerability, HFNOT might be a preferable option for these patients, as shown in a retrospective study by Koyauchi et al., in which 54 ILD patients with acute respiratory failure treated with HFNOT had significantly lower interruption and discontinuation rates than 30 patients treated with NIPPV (3.7% vs. 23.3%, *p* = 0.009, and 0% vs. 10%, *p* = 0.043, respectively) [73]. Current studies are based on retrospectively collected data; therefore, NIV is typically introduced when patients do not respond to COT. A key limitation is the lack of data directly comparing mortality in NIV and COT patients. There is an urgent need for RCTs to clarify the role of NIV in AE-ILD management.

#### 3.7.3. Lung Transplantation

Lung transplantation (LT) remains a viable therapeutic option for patients with IPF and is strongly recommended in selected cases. This intervention may be crucial for improving survival after AE. A retrospective study evaluated 108 patients with ILD, including 52 who experienced AE-ILD at the time of transplantation. The 90-day, 1-year, and 3-year survival rates after LT in patients with AE-ILD were 86.5%, 73.1%, and 60.1%, respectively, whereas the corresponding survival rates in patients without AE-ILD were 92.9%, 83.9%, and 79.6% (*p* = 0.032). The type of ILD (IPF vs. non-IPF ILD) did not significantly influence post-transplant outcomes [74]. Moreover, a recent meta-analysis including five cohort studies evaluated the prognosis of patients with AE-ILD (n = 183) who underwent LT, compared to those with stable ILD (n = 337), and found no significant differences in 90-day, 1-year, or 3-year survival between the two groups. Nevertheless, the length of hospitalization was significantly longer in the AE-ILD group. The authors concluded that the efficacy of LT in patients with AE-ILD is not inferior to that of patients with stable ILD [75].

#### 3.7.4. Real-World Practices in the Management of AE-ILD

The lack of guidelines and robust evidence for AE-ILD treatment leads to heterogeneous approaches, often based on individual physician experience. Kreuter et al. surveyed 509 pulmonologists from 66 countries to evaluate their clinical practices regarding the management of patients with AE-IPF. Most physicians routinely perform HRCT during the diagnostic process; however, the decision to perform BAL is based on clinical suspicion of infection. Treatment approaches are more diverse: 63% reported using 500–1000 mg methylprednisolone (or equivalent) for 3 days with tapering, while the second most common regimen was corticosteroids at a dose of 1 mg/kg/day, followed by slow tapering. Corticosteroids are used for an average of 13 weeks post-AE. Only 4% of the responders use alternative immunosuppressants for AE treatment. Broad-spectrum antibiotics combined with macrolides were routinely used by 56%, and 23% administered them based on clinical or laboratory indications. The majority of physicians reported continuing antifibrotic treatment during an AE episode. However, in patients not previously receiving such treatment, initiation of antifibrotics was often delayed until clinical stabilization. As preventive measures, most physicians prescribe vaccinations (93%), antifibrotic therapy (86%), pulmonary rehabilitation (58%), and anti-acid therapy (53%). Only 17% reported that elective surgery should be avoided; however, as many as 71% favored preventive anesthetic measures, such as low tidal volume ventilation and avoidance of hyperoxygenation, as well as the use of regional anesthesia over general anesthesia when feasible [76]. In the absence of data on effective treatments for AE-ILD, empirical experience in managing these patients remains essential for many physicians.

## 4. Conclusions

AE-ILD is a poorly understood, life-threatening complication of ILDs and is associated with an extremely poor prognosis. Additionally, the lack of standardized diagnostic criteria and unified management protocols increases diagnostic uncertainty and complicates subsequent clinical decision-making. There is no confirmed effective intervention for the management of AE-ILD. Current therapeutic strategies for AE-ILD are largely based on low-quality evidence, clinical experience, and expert opinion rather than high-quality data. Antifibrotic therapies have been associated with a reduced incidence of acute exacerbations and improved survival, underscoring the importance of maintaining patients on these treatments. Ultimately, AE-ILD remains a complex and largely unresolved clinical issue. There is an urgent need for standardized diagnostic criteria, evidence-based treatment protocols, and improved risk stratification. Further research, including prospective studies and clinical trials, is essential to advance understanding and treatment of AE-ILD. Until then, management will continue to rely on a case-by-case approach.

## Data Availability

No new data was created for this review.

## Abbreviations

The following abbreviations are used in this manuscript:

6MWT	6-minute walking test
AE	acute exacerbation
AIP	acute interstitial pneumonia
ALAT	Latin American Thoracic Association
ALI	acute lung injury
ALT	alanine aminotransferase
AMI	acute myocardial injury
ANA	antinuclear antibodies
ANCA	antineutrophil cytoplasmic antibodies
Anti-CCP	anti-cyclic citrullinated peptide antibodies
ARDS	Acute respiratory distress syndrome
AST	aspartate aminotransferase
ATS	American Thoracic Society
BNP	B-type natriuretic peptide
BAL	bronchoalveolar lavage
BP	blood pressure
bpm	beats per minute
CADM	clinically amyopathic dermatomyositis
CI	confidence interval
CK	creatine kinase
CMV	cytomegalovirus
CNI	calcineurin inhibitor
COT	conventional oxygen therapy
CPAP	continuous positive airway pressure
CRP	C-reactive protein
CT	computed tomography
CTD-ILD	connective tissue disease-associated interstitial lung disease
DAD	diffuse alveolar damage
DIC	disseminated intravascular coagulation
DLCO	diffusing capacity of the lung for carbon monoxide
DM	dermatomyositis
DM2	diabetes mellitus type
DNR	do-not resuscitate
EBV	Epstein-Barr virus
ERS	European Respiratory Society
EULAR	European Alliance of Associations for Rheumatology
FEV1	forced expiratory volume in 1 s
FiO_2_	fraction of inspired oxygen
ESC	European Society of Cardiology
FVC	forced vital capacity
GER	gastro-esophageal reflux
GGO	ground-glass opacities
HCO_3_	concentration of bicarbonate
HSP	heat shock protein
HFNOT	High-Flow Nasal Oxygen Therapy
HOT	home oxygen therapy
HP	hypersensitivity pneumonitis
HR	heart rate
HRCT	high-resolution computed tomography
HSV	herpes simplex virus
ICU	Intensive Care Unit
iDAD	idiopathic diffuse alveolar damage
IIM	inflammatory myopathy
IL-6	interleukin-6
IL-23	interleukin-23
ILD	interstitial lung disease
IMV	invasive mechanical ventilation
IIP	idiopathic interstitial pneumonia
IPs	interstitial pneumonias
IPF	idiopathic pulmonary fibrosis
IV	intravenous
IVIG	Intravenous Immunoglobulin
JAK	Janus kinase
JRS	Japanese Respiratory Society
LC	lung cancer
LDH	lactate dehydrogenase
LT	lung transplantation
MDA5	melanoma differentiation-associated gene 5
MDT	multidisciplinary team
MRSA	Methicillin-resistant *Staphylococcus aureus*
MOF	multi-organ failure
NIPPV	noninvasive positive pressure ventilation
NIV	non-invasive ventilation
NRM	non-rebreather mask
NSIP	nonspecific interstitial pneumonia
OP	organizing pneumonia
PAH	pulmonary artery hypertension
PaO_2_	arterial pressure of oxygen
pCO_2_	pressure of carbon dioxide
PCR	polymerase chain reaction
PCT	procalcitonin
PDE-4B	phosphodiesterase-4B
PDE-5	phosphodiesterase-5
PEEP	positive end-expiratory pressure
PET-CT	positron emission tomography-computed tomography
PF-ILD	progressive fibrosing interstitial lung disease
PH	pulmonary hypertension
PFTs	pulmonary function tests
PMX-DHP	direct hemoperfusion with polymyxin B
pO_2_	pressure of oxygen
PPF	progressive pulmonary fibrosis
RA	rheumatoid arthritis
RCTs	randomized controlled trials
RNA	ribonucleic acid
RP-ILD	rapid progressive interstitial lung disease
RR	relative risk
SAE	ubiquitin-like modifier activating enzyme
SCA	sudden cardiac arrest
SLB	surgical lung biopsy
SpO_2_	peripheral oxygen saturation
TGF-β	transforming growth factor-beta
TNF-α	tumour necrosis factor-alpha
TTV	torque teno virus
UIP	usual interstitial pneumonia
VC-AC	Volume Control-Assist Control
WBC	white blood cell
